# Paraneoplastic Sacroiliitis: A Masquerade of Inflammatory Arthritis in Leukemia

**DOI:** 10.7759/cureus.81650

**Published:** 2025-04-03

**Authors:** Ibad Sha, Nishanth P Kurian, Ibrahim S Majeed

**Affiliations:** 1 Department of Orthopedic Surgery, The Lifeline Multi Speciality Hospital, Adoor, IND; 2 Department of Orthopedics, The Lifeline Multi Speciality Hospital, Adoor, IND; 3 Department of Orthopedic Surgery, Mount Zion Medical College, Adoor, IND

**Keywords:** acute leukemia, axial arthritis, back pain, hematologic malignancy, inflammatory arthritis, leukemic arthritis, musculoskeletal oncology, paraneoplastic syndrome, sacroiliitis

## Abstract

Leukemic arthritis is a rare but clinically significant presentation of leukemia, often resembling inflammatory or autoimmune arthritis. Axial joint involvement, particularly sacroiliitis, is an uncommon manifestation and can mislead clinicians, delaying the diagnosis of the underlying hematologic malignancy. We present the case of a 34-year-old woman with persistent low back pain, initially diagnosed as sacroiliitis and treated with conventional therapies. Despite partial symptom relief with nonsteroidal anti-inflammatory drugs and corticosteroids, her condition worsened, leading to further evaluation. Subsequent laboratory tests revealed leukocytosis and anemia, and a peripheral blood smear confirmed leukemic infiltration. The final diagnosis of paraneoplastic sacroiliitis secondary to acute leukemia was established. This case highlights the importance of considering malignancy in patients presenting with refractory arthritis, particularly when symptoms are atypical or fail to respond to standard treatments. Early recognition and prompt initiation of chemotherapy can significantly improve clinical outcomes.​

## Introduction

Leukemic arthritis (LA) is an uncommon but well-recognized complication of leukemia, affecting approximately 4%-13% of adults and 12%-65% of children with leukemia [1]. It is frequently misdiagnosed as rheumatoid arthritis (RA), juvenile idiopathic arthritis (JIA), or seronegative spondyloarthropathies due to overlapping clinical features [1,2]. The most commonly involved joints are large peripheral joints such as the knees, ankles, and shoulders, while axial involvement, including sacroiliitis, is exceptionally rare [3]. Delayed recognition of LA can lead to unnecessary treatments, including prolonged corticosteroid therapy, which can negatively impact leukemia management.

Historically, the arthritis of leukemia was first described in the early 20th century, and subsequent studies have emphasized that musculoskeletal complaints may precede the hematologic diagnosis. The underlying pathophysiology remains multifactorial, involving direct leukemic cell infiltration into synovial tissue, immune-mediated synovitis, and hemorrhagic arthritis due to thrombocytopenia [3,4]. The diagnosis of LA can be difficult, especially in cases where initial blood counts and peripheral smears are normal, leading to a misclassification as a primary inflammatory arthritis.

While peripheral arthritis remains the most common presentation of LA, sacroiliitis as a paraneoplastic phenomenon is exceedingly rare. The presence of sacroiliac joint inflammation without systemic leukemic symptoms poses a significant diagnostic challenge, as clinicians often classify it under seronegative spondyloarthropathy. In this report, we describe a patient with paraneoplastic sacroiliitis due to LA, underscoring the importance of maintaining a high index of suspicion for malignancy in patients with refractory arthritis.

## Case presentation

A 34-year-old female schoolteacher presented with a 10-day history of progressively worsening low back pain. The pain was intermittent and dull, accompanied by early morning stiffness and partially relieved by rest. She denied any history of trauma, fever, weight loss, night sweats, or previous joint disease.

Clinical findings

On examination, bilateral sacroiliac joint tenderness was noted, which was more pronounced on the left side. There was no peripheral joint swelling or neurological deficits, and systemic examination was unremarkable. X-rays of the sacroiliac joints were normal, and initial laboratory tests showed a mildly elevated erythrocyte sedimentation rate (ESR; 18 mm/hour) with normal C-reactive protein (CRP; <5 mg/L) (Table 1). With a provisional diagnosis of inflammatory sacroiliitis, she was started on nonsteroidal anti-inflammatory drugs (NSAIDs) and gabapentin.

**Table 1:** Inflammatory markers showing elevated ESR with normal CRP levels ESR: erythrocyte sedimentation rate; CRP: C-reactive protein

Parameter	Patient value	Normal range
ESR	18	3-7 mm/hour
CRP	<5	<5 mg/L

Diagnostic assessment

**Figure 1:**
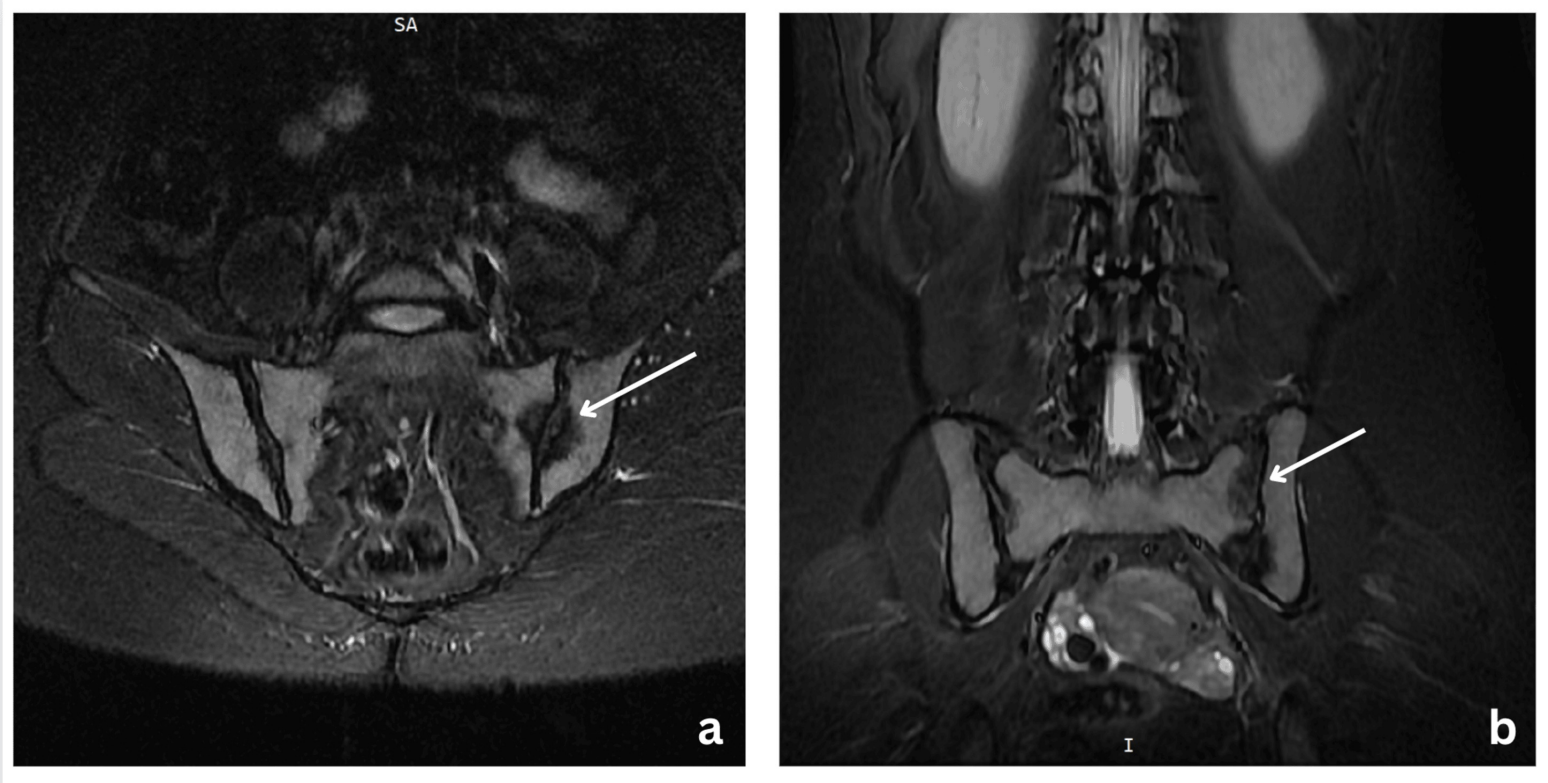
MRI images showing bilateral sacroiliitis. Axial (a) and coronal (b) short tau inversion recovery MRI sequences of the pelvis demonstrate bilateral sacroiliac joint inflammation characterized by hyperintense signal in the joint spaces and surrounding marrow edema. These findings are consistent with active sacroiliitis MRI: magnetic resonance imaging

After two weeks, the patient reported partial symptom relief (50%), leading to a tapering of her medications. However, one week later, the pain worsened significantly, along with difficulty walking. Concerned about persistent symptoms, a pelvis MRI was performed, revealing bilateral sacroiliitis (Figure 1). Given the worsening clinical picture, corticosteroid therapy was initiated, leading to a temporary improvement in symptoms.

Diagnosis and treatment

At her four-week follow-up visit, she developed severe itching unresponsive to antihistamines, prompting a repeat blood count. Laboratory reevaluation revealed hemoglobin of 9 g/dL and a total leukocyte count of 97,000/mm³ (Table 2). A peripheral blood smear demonstrated a predominance of atypical lymphoid cells (>90%) characterized by scant cytoplasm, high nuclear-to-cytoplasmic ratios, immature chromatin, and nuclear membrane irregularities, confirming a diagnosis of paraneoplastic sacroiliitis secondary to acute lymphoblastic leukemia (ALL). The patient was referred to hematology, where a bone marrow biopsy confirmed leukemia. The patient was initiated on the Berlin-Frankfurt-Münster protocol-based chemotherapy regimen for ALL, and within weeks, her sacroiliitis symptoms resolved, further establishing the paraneoplastic nature of her arthritis.

**Table 2:** Laboratory findings with normal ranges WBC: white blood cell; RBC: red blood cell; MCV: mean corpuscular volume; MCH: mean corpuscular hemoglobin; MCHC: mean corpuscular hemoglobin concentration; ESR: erythrocyte sedimentation rate; CRP: C-reactive protein; ALT: alanine transaminase; SGPT: serum glutamic-pyruvic transaminase; AST: aspartate transaminase; SGOT: serum glutamic-pyruvic transaminase

Parameter	Patient value	Normal range
Hemoglobin	9.8	12-16 g/dL
Total WBC count	97000	4,000-11,000/mm³
RBC count	2.96	4.2-5.4 million/mm³
Packed cell volume	27.6	38%-47%
Platelet count	2.25	1.5-4.5 lakh/mm³
Neutrophils	4.2	40%-60 %
Lymphocytes	89.6	20%-40%
Eosinophils	0	2%-8%
Monocytes	4.9	2%-6%
Basophils	1.3	0%-1%
MCV	93	80-96 fL
MCH	32.9	27-31 pg
MCHC	35.4	32-38 g/dL
ESR	18	3-7 mm/hour
CRP	<5	<5 mg/L
Albumin	4.5	3.4-4.8 g/dL
ALT/SGPT	29	0-31 U/L
AST/SGOT	16	0-32 U/L

## Discussion

LA can present in various ways, ranging from self-limited polyarthritis to persistent inflammatory arthritis mimicking RA or JIA [3-5]. It is commonly asymmetric, involving large joints, and may occur at any stage of leukemia. Axial involvement, particularly sacroiliitis, is extremely rare and can complicate timely diagnosis [5,6]. In this case, the absence of constitutional symptoms and localized presentation led to an initial diagnosis of primary inflammatory arthritis, delaying hematologic evaluation. Sacroiliitis as a manifestation of LA is unusual but should be considered, especially in atypical presentations of inflammatory arthritis. The presence of juxta-articular osteopenia, radiolucent bands, and metaphyseal lesions on X-ray or MRI should raise suspicion for a paraneoplastic process rather than primary inflammatory sacroiliitis [7]. In this patient, refractory sacroiliac joint inflammation without evidence of erosive disease was a key clue that prompted further hematologic evaluation.

The differential diagnosis of sacroiliitis in this patient included seronegative spondyloarthropathies, infectious sacroiliitis, reactive arthritis, and neoplastic infiltration [8,9]. Ankylosing spondylitis was unlikely due to the absence of human leukocyte antigen B27 positivity, while infectious sacroiliitis was ruled out based on negative cultures and lack of fever. Reactive arthritis was considered but deemed unlikely, as the patient had no history of recent infections or gastrointestinal symptoms. Ultimately, the presence of hematologic abnormalities and failure to respond to conventional arthritis treatments led to further investigation and the final diagnosis of LA secondary to leukemia.

The pathophysiology of LA is multifactorial and involves several proposed mechanisms [2,10,11]. One mechanism is the direct infiltration of leukemic cells into the synovial tissue, which leads to localized inflammation and joint symptoms. Additionally, immune complex deposition within the joint space can trigger immune-mediated synovitis, mimicking inflammatory arthritis. Another contributing factor is hemorrhagic arthritis, which results from leukemia-associated thrombocytopenia and leads to bleeding into the joint spaces. Finally, marrow expansion and periosteal irritation, particularly in bone-rich areas such as the sacroiliac joint, may contribute to pain and discomfort in affected patients.

Management of LA primarily focuses on treating the underlying leukemia, as joint symptoms typically resolve with effective chemotherapy. NSAIDs and corticosteroids may provide temporary symptom relief but do not alter disease progression [12]. A bone marrow biopsy and flow cytometry are essential for confirmatory diagnosis. Prognostically, early diagnosis and treatment significantly improve survival, but delayed recognition can result in complications, including joint damage and poor leukemia outcomes.

One important limitation in this case was the omission of a complete hemogram at the initial presentation. Given the patient’s young age and absence of systemic features, a focused workup was conducted, including inflammatory markers such as ESR and CRP. However, the lack of early hematological screening may have contributed to the delay in diagnosis. This highlights the importance of maintaining a high index of suspicion for systemic disease when inflammatory arthritis does not respond to standard therapy. In retrospect, a baseline hemogram, even in the absence of classical red flags, could have expedited the identification of leukemia and avoided unnecessary delay.

## Conclusions

This case highlights paraneoplastic sacroiliitis as a rare presentation of leukemia and underscores the importance of considering malignancy in patients with unexplained arthritis. While LA commonly affects peripheral joints, axial involvement is possible but rarely reported. Clinicians should remain vigilant for leukemia in cases of treatment-resistant arthritis, particularly when hematologic abnormalities emerge over time. Early diagnosis and prompt chemotherapy can lead to complete resolution of musculoskeletal symptoms and improved long-term outcomes.
